# Isolated Cardiac Rhabdomyoma With Marked ST-Segment Elevation: Benign Tumor, Malignant ECG

**DOI:** 10.1016/j.jaccas.2026.108875

**Published:** 2026-07-29

**Authors:** Devshankar Moosad EI, Gayathri Bhuvaneswaran Kartha, Anoop George Alex, Oommen K. George

**Affiliations:** Department of Cardiology, Christian Medical College, Vellore, India

**Keywords:** benign cardiac tumor, rhabdomyoma, ST-segment elevation

## Abstract

**Background:**

Cardiac rhabdomyoma is the most common primary cardiac tumor in infancy and is frequently associated with tuberous sclerosis complex (TSC).

**Case Summary:**

A 5-month-old asymptomatic infant presented with electrocardiography (ECG) showing marked ST-segment elevation in the anterolateral and inferior leads. Echocardiogram showed a cardiac rhabdomyoma in the left ventricle with preserved ventricular function and no evidence of hemodynamic compromise. Cardiac biomarkers were negative. There was no other evidence, clinically or radiologically, for TSC. Genetic testing for TSC was also negative. The infant remained clinically well with persistent but stable ECG changes at 10-month follow-up.

**Discussion:**

This case highlights a rare but striking ECG presentation of a benign cardiac tumor, emphasizing the importance of clinical context when interpreting ischemic-appearing ECG changes in infants.

**Take-Home Messages:**

Cardiac rhabdomyomas may present with marked ST-segment elevation on ECG, even in the absence of symptoms or myocardial injury. Ischemic-appearing ECG changes in infants with cardiac tumors may follow a benign clinical course and warrant careful longitudinal assessment rather than immediate intervention.

## Visual Summary

**Figure:**
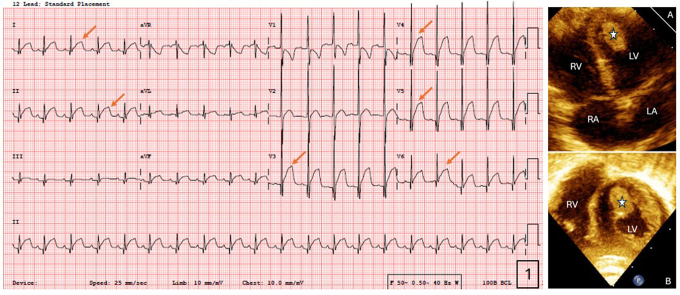

**Visual Summary:**
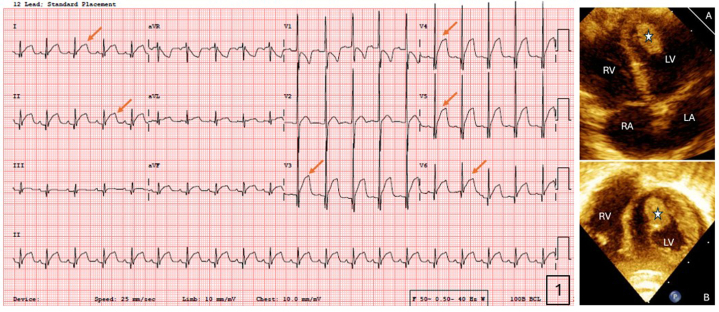
Rhabdomyoma With ST-Elevation Panel 1: ECG showing marked ST-segment elevation in the inferior leads (II, III, aVF) and the anterolateral leads (V_4_–V_6_), without reciprocal changes (indicated by orange arrows). (A and B) Echocardiographic images showing an isolated cardiac rhabdomyoma (indicated by white stars), seen as a well-circumscribed homogeneously echogenic structure seen attached to the anteroseptal aspect of LV apex. (A) Apical 4-chamber view. (B) Subcostal short-axis view at midcavity level. ECG = electrocardiogram; LA = left atria; LV = left ventricle; RA = right artery; RV = right ventricle.

## History of Presentation

A 5-month-old boy was referred for evaluation of a cardiac mass detected antenatally. He was asymptomatic, thriving well, and had no history of cyanosis, feeding difficulty, syncope, or arrhythmia. Cardiovascular examination was normal, with no murmurs or signs of heart failure. Apart from 4 small café-au-lait macules, there were no neurocutaneous stigmata. Ophthalmologic and neurologic examinations were unremarkable.

## Past Medical History

Fetal ultrasonography at 33 weeks' gestation identified 2 small intracardiac echogenic lesions, suspicious for cardiac rhabdomyomas. Delivery and the immediate neonatal course were uneventful apart from brief observation in the neonatal intensive care unit. There was no family history of tuberous sclerosis, cardiomyopathy, or sudden cardiac death.

Postnatal echocardiography had demonstrated 2 intracardiac masses: 1 attached to the interventricular septum near the left ventricular apex and a similar smaller mass in the right ventricular outflow tract. The infant was initially treated with sirolimus for approximately 3 months, resulting in complete regression of the right ventricular mass and significant reduction of the left ventricular mass. Sirolimus had to be stopped due to elevated liver transaminases. Subsequently, the tumor regressed completely. However, at 5-month follow-up, a similar mass had reappeared in the anteroseptal left ventricle with no notable hemodynamic consequence.

## Investigations

Echocardiography demonstrated a sessile, homogeneous echogenic mass attached to the interventricular septum near the left ventricular apex measuring 13 × 8 mm (Figure 1). Cardiac anatomy was otherwise normal, with preserved biventricular systolic function, unobstructed inflow and outflow tracts, and no pericardial effusion.

Electrocardiography (ECG) revealed marked ST-segment elevation in the inferior leads (II, III, aVF) and the anterolateral leads (V_4_–V_6_), without reciprocal changes (Figure 2).

The 24 hour Holter was negative for atrial and ventricular arrhythmias.

Cardiac biomarkers were negative.

Cardiac magnetic resonance imaging was deferred due to the young age and asymptomatic status.

Neuroimaging showed no cortical tubers, subependymal nodules, or subependymal giant cell astrocytomas.

Clinical exome sequencing did not identify pathogenic or likely pathogenic variants in *TSC1* or *TSC2.*

**Figure 1:**
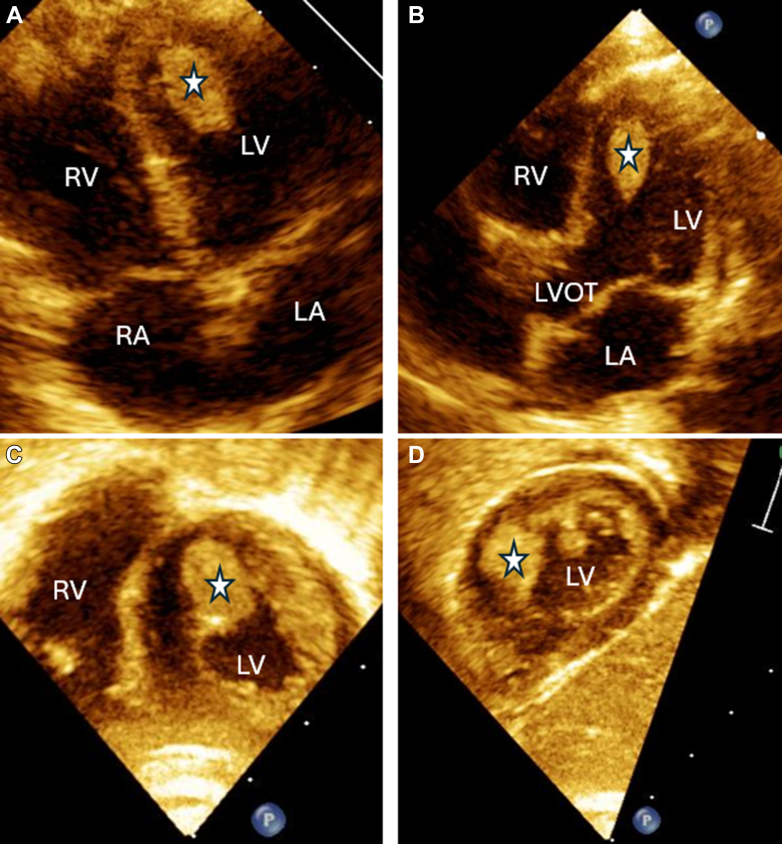
Echocardiographic Images Showing the Rhabdomyoma (Indicated by White Stars), Seen as a Well-Circumscribed Homogeneously Echogenic Structure Seen Attached to the Anteroseptal Aspect of LV Apex (A) Apical 4-chamber view, (B) apical 5-chamber view, (C) subcostal short-axis view at the midcavity level, (D) subcostal short-axis view at the level of LV apex. LA = left atria; LV = left ventricle; LVOT = left ventricular outflow tract; RA = right artery; RV = right ventricle.

**Figure 2:**
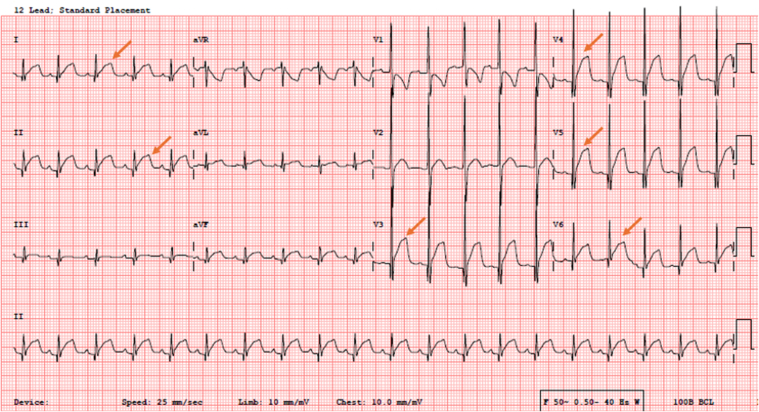
Electrocardiogram Showing Marked ST-Segment Elevation in the Inferior Leads (II, III, aVF) and the Anterolateral Leads (V_4_–V_6_), Without Reciprocal Changes (Indicated by Orange Arrows)

## Differential Diagnosis

Despite negative tuberous sclerosis complex (TSC) genetics, the diagnosis of cardiac rhabdomyoma was favored due to antenatal presentation, multiple fetal cardiac masses, typical echocardiographic appearance, and responsiveness to sirolimus therapy. Spontaneous tumor disappearance and reappearance is also typical for rhabdomyomas. Other primary cardiac tumors were considered less likely based on age, imaging characteristics, and clinical course.

## Management

As there was no evidence of hemodynamic compromise, the infant was managed conservatively.

## Outcome and Follow-Up

At 10-month follow-up, the infant remains clinically well with normal growth and development. The ST-segment elevation persists but remains stable. There is no evidence of ventricular dysfunction, myocardial injury, or arrhythmia.

## Discussion

Cardiac rhabdomyoma is the most common primary cardiac tumor in infancy and is strongly associated with TSC, reported in up to 80% to 90% of cases.^1^^,^^2^ Presence of multiple cardiac masses in a fetus is considered highly suggestive of rhabdomyoma and should typically prompt evaluation for TSC.^2^^,^^3^ However, isolated rhabdomyomas without clinical, radiologic, or genetic evidence of TSC have been described and represent a minority of cases.

ECG abnormalities in rhabdomyoma are well recognized. These include ventricular pre-excitation, repolarization changes, and atrial or ventricular arrhythmias and are likely related to tumor infiltration of the conduction system.^1^ Rarely, rhabdomyomas cause ST-segment elevation. Proposed mechanisms include myocyte compression, infiltration with fibrosis, and altered repolarization.^4^, ^5^, ^6^ A myopericarditis-like ECG pattern with concomitant ST elevation and PR-segment depression has also been described in infants with ventricular rhabdomyomas, and this has been attributed to pericardial stretch caused by large apical tumors. This pattern has been seen to improve on follow-up with tumor involution.^7^

Interestingly, among adults with cardiac masses, ST elevation on ECG has been shown to be more commonly associated with malignant cardiac tumors, particularly cardiac metastases. It is often accompanied by biomarker elevation and might represent myocardial invasion by tumor.^8^

Our case highlights that dramatic ischemic-appearing ECG changes can also occur in benign pediatric cardiac tumors without myocardial injury, underscoring the importance of integrating clinical status, biomarkers, and imaging before pursuing aggressive interventions.

## Conclusions

This case illustrates a rare presentation of isolated cardiac rhabdomyoma with persistent, marked ST-segment elevation in an asymptomatic infant without TSC. Recognition of this benign phenotype may prevent unnecessary invasive investigations or interventions and supports careful longitudinal follow-up.

## Funding Support and Author Disclosures

The authors have reported that they have no relationships relevant to the contents of this paper to disclose.

**Take-Home Messages**

• Cardiac rhabdomyomas may present with marked ST-segment elevation on electrocardiogram, even in the absence of symptoms or myocardial injury.
• Ischemic-appearing electrocardiographic changes in infants with cardiac tumors may follow a benign clinical course and warrant careful longitudinal assessment rather than immediate intervention.
• Negative genetic testing for tuberous sclerosis complex does not exclude the diagnosis of cardiac rhabdomyoma.
